# Ibuprofen is Not Associated With an Elevated Bleeding Risk or Transfusion Requirement in Skin Grafting for Patients With Burn Injuries

**DOI:** 10.1093/jbcr/iraf015

**Published:** 2025-02-26

**Authors:** Artur Manasyan, Jordan O Gasho, Michael I Kim, Eloise W Stanton, Maxwell B Johnson, T Justin Gillenwater

**Affiliations:** Keck School of Medicine, University of Southern California, Los Angeles, CA, United States; Keck School of Medicine, University of Southern California, Los Angeles, CA, United States; Keck School of Medicine, University of Southern California, Los Angeles, CA, United States; Division of Plastic and Reconstructive Surgery, Keck School of Medicine, Los Angeles, CA, United States; Division of Plastic and Reconstructive Surgery, Keck School of Medicine, Los Angeles, CA, United States; Division of Plastic and Reconstructive Surgery, Keck School of Medicine, Los Angeles, CA, United States

**Keywords:** ibuprofen, non-steroidal anti-inflammatory drugs, burn injury, skin graft

## Abstract

Ibuprofen, a non-steroidal anti-inflammatory drug, can increase the risk of bleeding, a significant concern in burn surgery, which often involves substantial blood loss. This study aims to evaluate the safety of ibuprofen use in burn patients undergoing skin grafting. A retrospective case-control chart review was conducted for patients admitted with acute burn injury from January 1, 2024 to July 31, 2024 who underwent skin grafting. The primary outcome variables included perioperative transfusion requirement, bleeding, skin graft failure, and other complications. A total of 53 patients met the inclusion criteria, 24 (45.2%) of whom received scheduled ibuprofen therapy during their hospitalization. The total body surface area affected was 12.3 ± 9.3% for the non-ibuprofen group and 14.3 ± 12.1% for the ibuprofen group (*P* = .62). A total of 79.3% of patients in the non-ibuprofen group received meshed grafts compared to 79.2% in the ibuprofen group (*P* = .734). Perioperative transfusion requirements were similar between the 2 cohorts, averaging 4.6 ± 3.1 for the non-ibuprofen group and 3.2 ± 2.8 units of packed red blood cells for the ibuprofen group (*P* = .207). Skin graft failure (defined as the need for re-grafting) occurred in 6.9% (*n* = 2) of the non-ibuprofen cohort vs none (*n* = 0) in the ibuprofen group (*P* = .112). Postoperative complications did not vary significantly between the 2 groups for seroma (*P* = .259), hematoma (*P* = .498), and infection (*P* = .568). There were no cases of hypersensitivity or gastrointestinal bleeding. There is likely no increased risk of bleeding or skin graft failure in burn injury patients taking ibuprofen, suggesting that these medications may be safe in this context.

## INTRODUCTION

Burns make up some of the most common injuries, with an estimated 9 million cases every year.^1,2^ While severity varies depending on the depth and extent of the injury, burn injuries necessitating inpatient admission are inevitably associated with pain during the acute burn period.^3–6^ Moreover, burns may result in chronic pain as a consequence of persistently activated nociceptive pathways due to nerve damage even the resolution of the acute injury.^7,8^ Pain, both in the acute and chronic form, can profoundly disrupt daily functioning and quality of life, requiring effective management strategies.^9^

Currently, most burn units utilize a combination of pharmacological and non-pharmacological interventions in acute pain management.^10,11^ The multimodal analgesia approach, which combines medications from different classes to target pain through multiple pathways, is often considered the most effective approach. This approach usually involves the use of acetaminophen, opioids, and neuropathic pain agents like gabapentin or pregabalin.^10,12,13^ Non-steroidal anti-inflammatory drugs (NSAIDs), by inhibiting the production of prostaglandins, can complement the analgesic effects of opioids and other medications, enhancing overall pain control in burn patients.^14–16^ Their utility lies primarily in targeting both the nociceptive and inflammatory pathways in conjunction with their generally favorable safety profile and availability.^14–16^ Ibuprofen, a specific type of NSAID, is most commonly used by the general population for its effectiveness in relieving pain and reducing inflammation.^17^

Nevertheless, ibuprofen is not without potential risks, especially concerning bleeding, a significant concern in burn surgery, which often involves substantial blood loss. By inhibiting the cyclooxygenase (COX) enzyme, platelet aggregation and vasoconstriction are disrupted, impairing platelet function, and clotting ability.^14–17^ While effective in managing pain, in burn patients, where surgical interventions and skin graft procedures are common, this heightened bleeding risk poses significant concerns postoperatively. Impaired hemostasis may increase the likelihood of intraoperative bleeding and compromise skin graft adherence due to surgical site bleeding, highlighting the need for a careful risk-benefit assessment in this specific patient population.^18^

As such, in this study, we seek to assess bleeding risk in patients with burn injuries undergoing skin grafting. By evaluating the safety profile of ibuprofen in this specific context, we hope to provide valuable insights that can help optimize postoperative care, minimize complications, and improve patient outcomes while effectively managing pain.

## METHODS

### Inclusion and exclusion criteria

Inclusion criteria were as follows: adult (18+ years of age) patients presenting with acute burn injury of 5%–20% total body surface area (TBSA) to our burn unit from January 1, 2024 to July 31, 2024 who underwent skin grafting surgery. The ibuprofen protocol consisted of administering 600 mg of scheduled ibuprofen therapy every 6 h. Patients were included in the ibuprofen cohort only if they received ibuprofen for at least 48 h before and after surgery; those on PRN or 1-time doses were excluded. The control cohort did not receive ibuprofen or any other NSAIDs, primarily utilizing opioids and acetaminophen for pain management. Patients under 18 years of age or with significant comorbidities (ie, congestive heart failure, chronic kidney disease) were excluded. Patients with any history of bleeding/clotting disorder, including, but not limited to, von Willebrand disease, hemophilia A, hemophilia B, thrombocytopenia, Factor V Leiden, protein C deficiency, protein S deficiency, and antiphospholipid syndrome were also excluded.

### Data analysis

Data collection was conducted for the following variables: age, sex, race/ethnicity, burn etiology, affected TBSA, alcohol/drug use, comorbidities, surgical history, inpatient length of stay, length of ventilator support, length of intensive care unit stay, burn surgery records, and postoperative complications (eg, hematoma, gastrointestinal bleeding, etc.). The primary outcome variables included perioperative transfusion requirement, bleeding, skin graft failure, and other complications. Descriptive statistics, Welch’s *t*-test of unequal variances, and chi-squared analysis were performed using Microsoft Excel version 16 (Redmond, WA: Microsoft Corporation). A *P*-value of .05 was set as statistically significant.

## RESULTS

### Patient characteristics

A total of 53 patients met the inclusion criteria, 24 (45.2%) of whom received scheduled ibuprofen therapy during their hospitalization for acute burn injury. The mean age was 46.3 ± 20.8 and 43.7 ± 13.6 years in the control and ibuprofen cohorts (0.591), respectively. There was no statistically significant difference in sex (65.5%, *n* = 19 vs 83.3%, *n* = 20, *P* = .142) or race (0.231). The TBSA affected was 12.3 ± 9.3% for the non-ibuprofen group and 14.3 ± 12.1% for the ibuprofen group (*P* = .62). In the non-ibuprofen group, 10 (34.5%) experienced second-degree burns and 19 (65.6%) experienced third-degree burns, while in the ibuprofen group, 7 (29.2%) had second-degree burns and 17 (70.8%) had third-degree burns (0.737). A total of 79.3% of patients in the non-ibuprofen group received meshed grafts compared to 79.2% in the ibuprofen group (*P* = .734). Patient characteristics are presented in Table 1.

### Outcomes

The operative time was 167.0 ± 95.9 min for the non-ibuprofen group and 148.1 ± 59.3 min for the ibuprofen group, with no significant difference (*P* = 0.405). Patients received ibuprofen for an average of 3.4 ± 1.8 days before surgery and 3.6 ± 2.1 days postoperatively. Hematoma occurred once in the non-ibuprofen group (3.4%) and once in the ibuprofen group (4.2%) (*P* = .498). Specifically, the 2 cases of hematoma occurred in patients who received meshed grafts, with no cases of hematoma in the meshed cases. Perioperative transfusion requirements were similar between the 2 cohorts, averaging 4.6 ± 3.1 for the non-ibuprofen group and 3.2 ± 2.8 units of packed red blood cells for the ibuprofen group (*P* = .207). Skin graft failure (defined as the need for re-grafting) occurred in 6.9% (*n* = 2, 1 meshed and 1 non-meshed) of the non-ibuprofen cohort vs none (*n* = 0) in the ibuprofen group (*P* = .112). Postoperative complications did not vary significantly between the 2 groups for seroma (*P* = .259) and infection (*P* = .568). There were no cases of hypersensitivity or gastrointestinal bleeding. Length of inpatient stay was an average of 20.17 days for the non-ibuprofen group vs 15.9 days for the ibuprofen group (*P* = .631). Outcomes are presented in Table 2.

## DISCUSSION

The use of NSAIDs in patients undergoing invasive procedures like burn surgery has been approached with caution due to concerns of increased bleeding risk.^19–21^ Our study sought to address the gap in evidence surrounding the safety of NSAIDs specifically in burn surgery by evaluating their impact on bleeding risk and perioperative outcomes. In our cohort, we found no significant differences in operative time, hematoma formation, transfusion requirements, or postoperative complications between the ibuprofen and non-ibuprofen groups. These findings challenge the assumption that NSAIDs increase bleeding risk in the burn surgery patient population and suggest that they may have a role in multimodal pain management.

The absence of increased bleeding risk observed in our study may be attributed to the specific action of ibuprofen on COX enzymes. Unlike anticoagulants like aspirin, which irreversibly inhibit COX-1 and affect platelet aggregation, ibuprofen reversibly inhibits COX-1, causing only a temporary reduction in platelet function.^22^ This reversible inhibition may explain the lack of increased bleeding risk that we observed in our study. Additionally, NSAIDs have a lesser impact on homeostasis, which may further reduce their potential to increase bleeding risk.^22^ While no other studies have specifically examined NSAIDs in skin grafting for burn patients, research on other surgical procedures collectively indicates that NSAIDs do not increase perioperative bleeding, as also corroborated by our study.^23^ In the context of skin grafting, meshed skin grafts allow for better drainage of exudates and blood, reducing the risk of hematoma formation underneath the graft. In contrast, non-meshed grafts have a higher tendency for hematoma accumulation due to the lack of drainage pathways, potentially leading to graft failure. While our study’s small sample size precluded definitive conclusions, future research should focus on the comparison of meshed vs non-meshed grafts, particularly in contexts where hematoma formation is a significant concern, as in the use of NSAIDs for pain management.

The use of opioids in burn care has been a cornerstone of pain management, particularly given the chronic and often neuropathic nature of burn pain.^24^ However, burn patients are at increased risk of opioid addiction due to the often prolonged nature of their pain and the frequent need for extended opioid therapy.^25^ This risk highlights the importance of exploring alternative or adjunctive therapies that can mitigate opioid dependency. Our study demonstrated that the use of ibuprofen did not have adverse effects on perioperative and postoperative outcomes. These findings suggest that ibuprofen can be safely integrated as part of a multimodal pain management strategy for burn patients, while potentially reducing opioid requirements and minimizing the risk of addiction for this patient population.

One intriguing aspect is the potential role of ibuprofen beyond its traditional anti-inflammatory and analgesic properties in burn surgery. Specifically, their anti-inflammatory effects may help mitigate the systemic inflammatory response seen in severe burns, potentially influencing wound healing dynamics, and reducing the incidence of complications such as hypertrophic scarring.^26^ This dual benefit—pain relief coupled with potential modulation of the inflammatory cascade—presents a compelling case for reevaluating ibuprofen use in burn care protocols. Only 1 study reported on the use of topical ibuprofen in the context of wound healing, which demonstrated that while it provided effective analgesia, there was merely no negative effect on healing.^27^ However, NSAIDs, by inhibiting COX-1 and COX-2, reduce the production of prostaglandins, which play a crucial role in the inflammatory phase of wound healing and reducing scarring. This reduction in prostaglandins leads to decreased fibroblast proliferation, collagen synthesis, and expression of matrix metalloproteinases, which are essential in regulating the balance between extracellular matrix (ECM) synthesis and degradation during the healing process. Nevertheless, this relationship between NSAIDs and the theoretical implications in burn wound healing is very limited, with no studies focusing on oral or intravenous agents. Thus, this topic requires further investigation to more comprehensively understand the role of NSAIDs in the pain management of patients with burn injuries.

While ibuprofen shows promise in burn management management, its potential side effects must be carefully considered, particularly in burn patients. Gastrointestinal bleeding is a known complication of NSAID use, and this risk may be exacerbated in patients with significant stress responses, such as those with major burn injuries.^28,29^ Additionally, NSAIDs can impair kidney function, which is of particular concern in burn patients who may already be at risk for acute kidney injury due to fluid shifts and systemic inflammation.^30^ Therefore, the decision to use ibuprofen in burn management must be made in consideration of these potential risks and should involve monitoring of gastrointestinal health and renal function.

This study provides valuable insight into the use of ibuprofen in patients with acute burn injury undergoing surgery, but there are some limitations. The most notable was that our sample size was small, reducing the power of the statistical analysis, in addition to the retrospective nature of the study. Another notable limitation is that we did not collect patient-reported outcomes on pain control, which could provide important insights into the experience of pain relief and overall satisfaction with ibuprofen use amongst burn patients. Future randomized controlled trials should aim to include patient-reported outcomes to better understand the efficacy of ibuprofen, among other NSAIDs, in managing pain in burn patients.

Given the novelty of this research topic, further research is needed to confirm the bleeding risk of ibuprofen specifically in the burn care context, as well as their potential beneficial effects on wound healing. Such research can potentially lead to an evidenced-based integration of ibuprofen more broadly in burn care protocols as part of a multimodal approach to pain and inflammation management.

## CONCLUSION

Our findings support the potential use of ibuprofen in burn injury management without heightened concern for bleeding or graft complications, although the literature remains limited to make conclusive claims. Future research should investigate the effects of other NSAID agents and involve larger, randomized controlled trials to support these findings.

**Table 1.** Patient and Burn Characteristics in the Non-NSAID vs NSAID Cohorts

Variable	Non-NSAID (*n* = 29)	NSAIDs (*n* = 24)	*P*-value
Age	46.3 + 20.8	43.7 + 13.6	.591
Male (*n*, %)	19 (65.5)	20 (83.3)	.142
Race			.231
White	4 (13.8%)	3 (12.5%)	
Black	6 (20.7%)	4 (16.7%)	
Hispanic	13 (45%)	10 (41.7%)	
Asian	1 (3.5%)	1 (4.2%)	
Other	5 (17.2%)	6 (24.9%)	
TBSA	12.3 + 9.3	14.3 + 12.1	.62
Burn degree			.737
1st	0 (0)	0 (0)	
2nd	10 (34.5)	7 (29.2)	
3rd	19 (65.6)	17 (70.8)	
Operative time	167.0 + 95.9	148.1 + 59.3	.405
Skin graft type			.734
Meshed	23 (79.3)	19 (79.2)	
Sheet	6 (20.7)	5 (20.8)	

**Table 2.** Postoperative Outcomes in the Non-NSAID vs NSAID Cohorts

Variable	Non-NSAID (*n* = 29)	NSAIDs (*n* = 24)	*P*-value
Infection	2 (6.9%)	1 (4.17%)	.568
Hematoma	1 (3.4%)	1 (4.2%)	.498
Seroma	3 (10.3%)	1 (4.17%)	.259
Hypersensitivity	0 (0%)	0 (0%)	1.0
Gastrointestinal bleed	0 (0%)	0 (0%)	1.0
Skin graft failure	2 (6.9%)	0 (0%)	.112
Dehiscence	0 (0%)	0 (0%)	1.0
Transfusion requirement	13 (44.8%)	10 (41.67%)	.75
Mean transfusion units	4.6 ± 3.1	3.2 ± 2.8	.207
Length of stay (days)	20.2	15.9	.631
